# A Novel Gene Signature-Based Model Predicts Biochemical Recurrence-Free Survival in Prostate Cancer Patients after Radical Prostatectomy

**DOI:** 10.3390/cancers12010001

**Published:** 2019-12-18

**Authors:** Run Shi, Xuanwen Bao, Joachim Weischenfeldt, Christian Schaefer, Paul Rogowski, Nina-Sophie Schmidt-Hegemann, Kristian Unger, Kirsten Lauber, Xuanbin Wang, Alexander Buchner, Christian Stief, Thorsten Schlomm, Claus Belka, Minglun Li

**Affiliations:** 1 Department of Radiation Oncology, University Hospital, LMU Munich, D-81377 Munich, Germany; 2 Institute of Radiation Biology, Helmholtz Center Munich, German Research Center for Environmental Health, D-85764 Neuherberg, Germany; 3 Technical University of Munich, D-80333 Munich, Germany; 4 Biotech Research & Innovation Centre (BRIC) and Finsen Laboratory, University of Copenhagen, Rigshospitalet, DK-2200 Copenhagen, Denmark; 5 Charité Universitätsmedizin Berlin, Charité platz 1, D-10117 Berlin, Germany; 6 Research Unit Radiation Cytogenetics, Helmholtz Center Munich, German Research Center for Environmental Health GmbH, D-85764 Neuherberg, Germany; 7 Clinical Cooperation Group ‘Personalized Radiotherapy in Head and Neck Cancer’, Helmholtz Zentrum München, German Research Center for Environmental Health GmbH, D-85764 Neuherberg, Germany; 8 Laboratory of Chinese Herbal Pharmacology, Oncology Center, Renmin Hospital, Hubei University of Medicine, Shiyan 442000, China; 9 Department of Urology, University Hospital, LMU Munich, D-81377 Munich, Germany; 10 Martini-Clinic Prostate Cancer Center at the University Medical Center Hamburg-Eppendorf, D-20246 Hamburg, Germany

**Keywords:** prostate cancer, radical prostatectomy, gene signature, risk stratification, biochemical recurrence-free survival

## Abstract

Currently, decision-making regarding biochemical recurrence (BCR) following prostatectomy relies solely on clinical parameters. We therefore attempted to develop an integrated prediction model based on a molecular signature and clinicopathological features, in order to forecast the risk for BCR and guide clinical decision-making for postoperative therapy. Using high-throughput screening and least absolute shrinkage and selection operator (LASSO) in the training set, a novel gene signature for biochemical recurrence-free survival (BCRFS) was established. Validation of the prognostic value was performed in five other independent datasets, including our patient cohort. Multivariate Cox regression analysis was performed to evaluate the importance of risk for BCR. Time-dependent receiver operating characteristic (tROC) was used to evaluate the predictive power. In combination with relevant clinicopathological features, a decision tree was built to improve the risk stratification. The gene signature exhibited a strong capacity for identifying high-risk BCR patients, and multivariate Cox regression analysis demonstrated that the gene signature consistently acted as a risk factor for BCR. The decision tree was successfully able to identify the high-risk subgroup. Overall, the gene signature established in the present study is a powerful predictor and risk factor for BCR after radical prostatectomy.

## 1. Introduction

Prostate cancer (PCa) is the second most commonly diagnosed cancer in men worldwide [1]. Over half of PCa patients will undergo radical prostatectomy as their primary treatment choice [2]. After radical prostatectomy, approximately 20% of patients experience a biochemical recurrence (BCR) with a rising prostate-specific antigen (PSA) level [3]. Several randomized phase III trials have shown that adjuvant radiotherapy is beneficial for patients with high-risk factors such as pathological T3/4 (pT3/4) or R1 resection status [4,5,6]. However, about 50% of these patients did not suffer a biochemical recurrence without adjuvant radiotherapy, even after a long follow-up of 5 years [7]. For these patients, adjuvant radiotherapy would be an overtreatment, with some risk of unnecessary radiation-induced side effects. Hence, a more precise method to identify patients suffering BCR after radical prostatectomy is a critical issue for the optimal management of PCa.

Nowadays, advancements in high-throughput techniques such as microarray and RNA-sequencing (RNA-seq) have provided new insights into transcriptome profiling, which facilitate the utilization of molecules as diagnostic and prognostic biomarkers [8,9]. Some studies have established gene signatures to help distinguish aggressive PCa tumors or improve survival prediction in PCa patients [10,11,12]. However, most of these signatures exhibit a prognostic value without having a direct impact on treatment decision-making.

In this study, we established a gene expression-based signature to improve the prediction of BCR after radical prostatectomy, using a univariate and least absolute shrinkage and selection operator (LASSO) Cox model. Then, the prognostic value of the gene signature was further validated in five independent datasets across multiple platforms and our patient cohort. As regards clinical application, the gene signature was combined with clinicopathological features to build a decision tree to improve risk stratification for BCR. In addition, bioinformatic analyses were performed to reveal the biological processes and potential pathways underlying the gene signature.

## 2. Methods

### 2.1. Dataset Preparation and Sample Collection

In total, 903 PCa samples with full-scale clinical annotations (age, Gleason score, pathological T stage, surgical margin status, and follow-up BCR information) from six independent cohorts were included in our study. Three cohorts were from Gene Expression Omnibus (GEO), one cohort from The Cancer Genome Atlas (TCGA), one cohort from Memorial Sloan Kettering Cancer Center (MSKCC, Manhattan, NY, US), and our patient cohort was collected from University Medical Center Hamburg-Eppendorf, Germany (Hamburg, Germany). GSE70769 and GSE70768 were involved in the same research [13], and the microarray data were produced with the same chip platform (Illumina HumanHT-12 V4.0 Array). The RNA-seq data of GSE54460 were produced with Illumina HiSeq 2000, for 94 patients with full-scale clinical records [14]. Additionally, RNA-seq data of 388 patients were accessed from TCGA, and microarray data of 138 patients (produced with Affymetrix Human Exon 1.0 ST Array) were obtained from MSKCC [15]. Probe IDs were mapped to gene symbols according to the corresponding annotation file, and expression measurements of all probes linking to the same gene were averaged to obtain a single value. Finally, samples from 84 patients were consecutively collected at the Department of Urology and the Martini Clinics at the University Medical Center Hamburg-Eppendorf (UKE) from 2010 to 2016. Informed consent and an ethical vote in Ethics Commission University Hamburg (ethic codes WF-049/09 and PV3652) were obtained according to the current International Cancer Genome Consortium (ICGC) guidelines (see http://www.icgc.org). Written informed consent was obtained from each patient as described in our previous study [16]. GSE70769 was used as a training set, while the other five cohorts were used to validate. All microarray and RNA-seq data in our study were normalized and log2 transformed, and expression measurements of multiple samples taken from the same patient were averaged to a single value.

### 2.2. Candidate Selection and Signature Establishment

The weighted gene co-expression network analysis (WGCNA) R package [17] was used to construct a scale-free co-expression network based on the microarray data of the training cohort. The weighted network adjacency was defined by the formula $a_{i,j}=s_{i,j}^{\beta }\;$, $s_{i,j}=\left|cor\left(x_{i},x_{j}\right)\right|\;$. (*x_i_*, *x_j_*: each pair of genes; cor: Pearson’s correlation; β: soft-power threshold). The topological overlap matrix (TOM) was constructed based on the adjacency, and the corresponding dissimilarity (1-TOM) was used as the distance measure, with deepSplit of 2 and minModuleSize of 30, to assign whole-genome genes into different modules via hierarchical clustering analysis. Unassigned genes were categorized into the gray module. Gene significance quantifies the association of individual genes with biochemical recurrence-free survival (BCRFS) status, and module membership represents the correlation between the module eigengene and the gene expression profile. Among non-gray modules, the modules which had the highest absolute correlations with BCRFS status were selected as candidate modules for further selection. Genes from these modules were submitted for high-throughput univariate Cox regression analysis to screen for prognostic candidates. Subsequently, the LASSO Cox regression model was used to further screen for the most robust prognostic markers [18]. Finally, a risk score (RS) formula was established with individual normalized gene expression values weighted by their LASSO Cox coefficients as follows: $\underset{i}{\sum }Coefficient\left(mRNA_{i}\right)\times Expression\left(mRNA_{i}\right)$.

### 2.3. Bioinformatic Analyses

WGCNA was used to construct a scale-free co-expression network and to identify the most significant modules, with a risk score based on TCGA RNA-seq data. Hub genes with gene significance >0.3 in the black module were extracted and submitted for Kyoto Encyclopedia of Genes and Genomes (KEGG) enrichment analysis, and a Circos diagram was used to visualize outputs [19]. Moreover, gene set enrichment analysis (GSEA) [20] was performed to analyze the potential signaling pathways underlying the gene signature, using gene set “hallmark.all.v6.1.symbols.gmt”, based on TCGA RNA-seq data.

### 2.4. Statistical Analyses

IBM SPSS Statistics version 20 (IBM Corp., Armonk, NY, USA), GraphPad Prism 8.0 (GraphPad Software Inc., San Diego, CA, USA), Stata 12 (StataCorp LLC, College Station, TX, USA) and R software (version 3.5.2, http://www.r-project.org) were used to analyze data and plot graphs. The Kaplan–Meier method was applied to draw survival curves, and the log-rank test was used to evaluate survival difference. The Cox proportional-hazards regression model was used to evaluate the significance of each parameter for biochemical recurrence-free survival (BCRFS). Time-dependent receiver operating characteristic (tROC) analysis was performed to measure the predictive power, using the “survivalROC” package [21], and areas under the curve (AUC) of each variable at different time nodes were compared. Meta-analysis (I^2^ < 30%, fixed-effect model) was performed to evaluate the prognostic value in the pooled cohort. The Z-score method was used to normalize risk scores in each cohort. Recursive partitioning analysis (RPA) was performed to construct decision trees using the ”rpart” package [22]. Student’s t-test or one-way analysis of variance was used to analyze differences between groups in variables with a normal distribution.

## 3. Results

### 3.1. Establishment of a Prognostic Gene Signature for BCRFS

**Figure 1:**
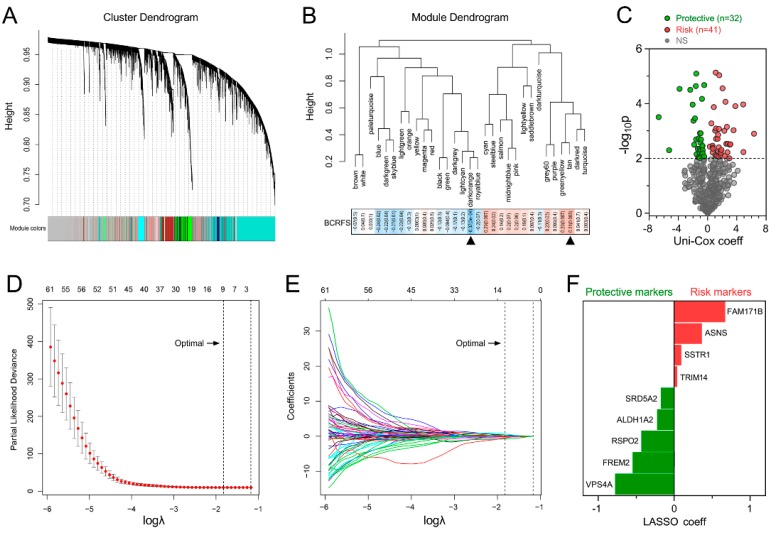
Selection of robust biomarkers to establish a prognostic gene signature. (**A**) Weighted gene co-expression network analysis (WGCNA) was performed to construct a scale-free network, and whole-genome genes from the training cohort were assigned to different modules. (**B**) Two modules (darkorange and tan) were mostly correlated with biochemical recurrence (BCR), and 455 candidates were extracted for further study. (**C**) Univariate Cox regression analysis was performed to screen for significant candidates. (**D**) Cross-validation was applied to prevent overfitting, and an optimal λ value of 0.1614 with log(λ) = −1.8239 was selected. (**E**) Nine genes finally remained with their nonzero LASSO coefficients. (**F**) Distribution of least absolute shrinkage and selection operator (LASSO) coefficients of the gene signature.

First, WGCNA was performed with microarray data and BCRFS status on the training cohort. Sample clustering showed that no outlier was detected (Figure S1). With a power of β = 2 set as the optimal soft threshold to construct a scale-free network, a total of 31 non-grey modules were identified (Figure 1A). Among these non-grey modules, two modules (darkorange and tan) with the highest absolute correlation values with BCRFS were picked out (Figure 1B). Then, 455 genes from these two modules were submitted for high-throughput univariate Cox regression analysis. With a threshold of *p* < 0.01, 73 promising candidate genes (32 protective and 41 risk markers) were identified (Figure 1C). Next, the LASSO Cox regression model was used to identify robust markers among the 73 candidates. Cross-validation was applied to prevent overfitting, and the optimal λ value of 0.1614 with log(λ) = −1.8239 was selected (Figure 1D). Nine genes (*ALDH1A2*, *ASNS*, *SSTR1*, *FAM171B*, *FREM2*, *RSPO2*, *SRD5A2*, *TRIM14*, and *VPS4A*) remained with their individual nonzero LASSO coefficients (Figure 1E). The distribution of LASSO coefficients of the gene signature is demonstrated in Figure 1F. Finally, the risk score (RS) of the gene signature was established as follows:

Risk score = (−0.22345 * expression level of *ALDH1A2*) + (0.364318 * expression level of *ASNS*) + (0.67184 * expression level of *FAM171B*) + (−0.54351 * expression level of *FREM2*) + (−0.4304 * expression level of *RSPO2*) + (−0.17707 * expression level of *SRD5A2*) + (0.094559 * expression level of *SSTR1*) + (0.040268 * expression level of *TRIM14*) + (−0.77555 * expression level of *VPS4A*).

The expression levels of each gene were log2 normalized. Additionally, the expression profiles of the gene signature were mainly dysregulated across 497 tumors and 52 adjacent normal tissues from the TCGA data (Figure S2).

### 3.2. Gene Signature Serves as a Risk Factor and Promising Predictor for BCRFS

**Figure 2:**
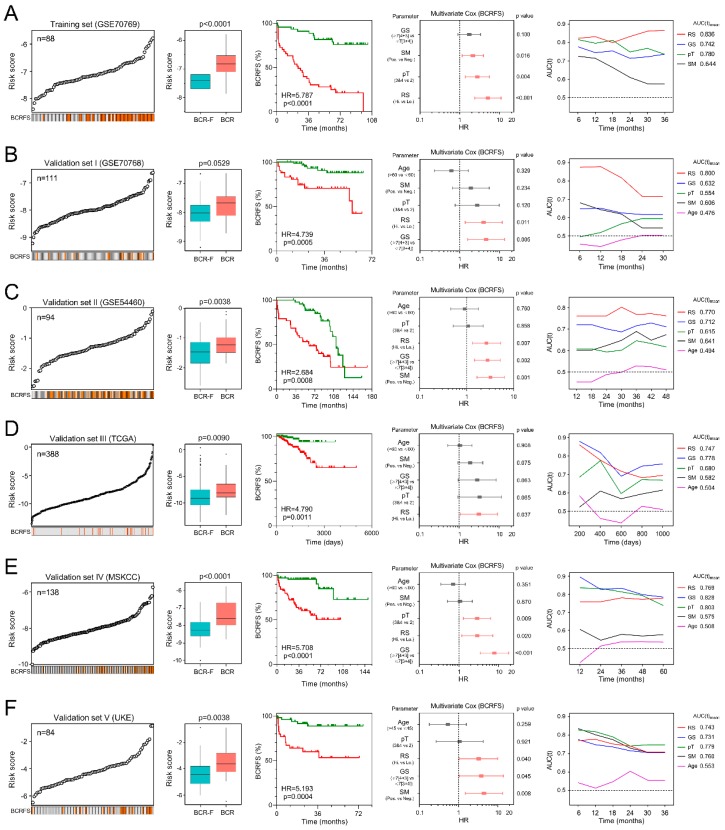
Gene signature serves as a risk factor and promising predictor for biochemical recurrence-free survival (BCRFS) in each cohort. (**A**–**F**) In each cohort, the risk score was significantly elevated in BCR patients compared with BCR-free (BCR-F) ones. Kaplan–Meier analysis showed patients with higher scores exhibited a worse prognosis. The multivariate Cox regression model indicated that the risk score was an independent risk factor for BCRFS in each cohort. Time-dependent receiver operating characteristic (ROC) analysis showed the risk score was a powerful and stable predictor for BCR in each cohort.

We ranked the risk scores of all patients in the training cohort, and the risk scores of BCR patients were significantly elevated compared with those of BCR-free (BCR-F) ones. Kaplan–Meier survival analysis demonstrated that the two groups exhibited significantly different outcomes (Hazard Ratio (HR) = 5.787, *p* < 0.0001). Multivariate Cox regression analysis showed that the risk score was an independent risk factor for BCRFS (HR = 5.084, *p* < 0.0001). tROC analysis indicated that the risk score also functioned as a powerful predictor for BCR, with an average AUC(t) of 0.836 at 36 months follow-up (Figure 2A).

To confirm the prognostic robustness of the gene signature, it was further validated in five other independent cohorts (Figure 2B–F). Consistently, patients with higher risk scores exhibited significantly worse BCRFS than patients with lower risk scores in Kaplan–Meier analysis in all five cohorts (validation I: HR = 4.739, *p* = 0.0005; validation II: HR = 2.684, *p* = 0.0008; validation III: HR = 4.790, *p* = 0.0011; validation IV: HR = 5.708, *p* < 0.0001; validation V: HR = 5.193, *p* = 0.0004). Furthermore, multivariate Cox regression analysis was performed on the risk score and clinicopathological features including age, Gleason score (GS), pathological T stage (pT) and surgical margin (SM), to evaluate the significance of each for BCR risk. Notably, the risk score was always an independent risk factor for BCRFS in all five validation series (validation I: HR = 3.979, *p* = 0.011; validation II: HR = 2.616, *p* = 0.007; validation III: HR = 3.120, *p* = 0.037; validation IV: HR = 2.913, *p* = 0.020; validation V: HR = 3.241, *p* = 0.040). tROC analysis demonstrated that the risk score exhibited the most powerful prediction in validations I and II, while having similar predictive power to some clinicopathological parameters such as Gleason score or pT in validations III, IV, and V. Interestingly, among all the clinical variables, age was neither a risk factor nor a promising predictor for BCR in all five validation cohorts.

**Figure 3:**
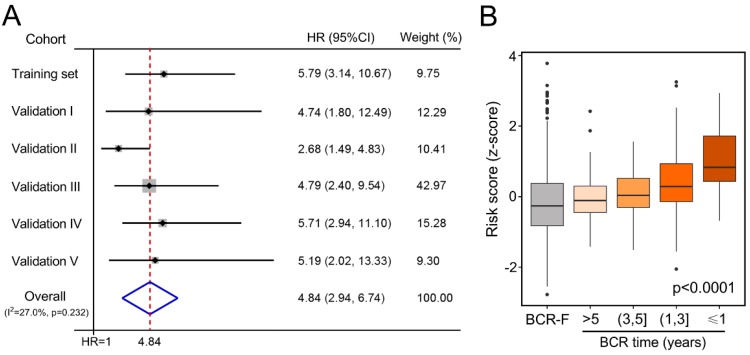
Gene signature-derived risk score could identify high-risk patients in the pooled cohort. (**A**) Meta-analysis indicated that patients with higher risk scores exhibited worse prognosis compared to those with lower ones (HR = 4.84, 95% CI = 2.94–6.74) in the pooled cohort. Additionally, risk scores were normalized to Z-scores in each cohort, and we observed that (**B**) Z-scores of risk scores were significantly elevated in BCR patients compared with BCR-free (BCR-F) patients, especially in shorter-term BCR patients.

Next, meta-analysis was used to analyze the prognostic value of the gene signature in the pooled cohort. Our result indicated that a higher risk score was correlated with a significantly worse prognosis in the pooled cohort (HR = 4.84, 95% CI = 2.94–6.74; Figure 3A). Additionally, we normalized risk scores to Z-scores for each cohort and found that Z-scores were significantly elevated in BCR patients compared to BCR-free (BCR-F) patients. Further, Z-score was more sensitive for the prediction of an early biochemical recurrence, as demonstrated in Figure 3B (*p* < 0.0001).

### 3.3. Combination with Clinical Variables to Improve Risk Stratification

**Figure 4:**
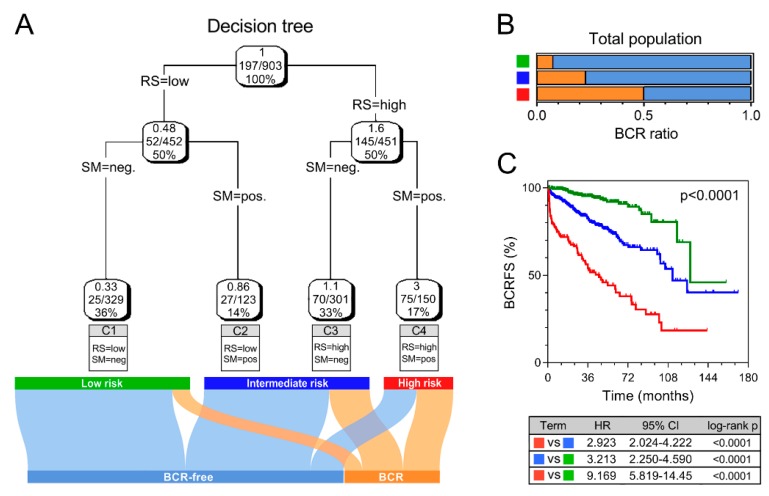
Combination of risk score and clinicopathological features to improve risk stratification and survival prediction. (**A**) A decision tree was generated to optimize risk stratification in the pooled cohort, and risk score served as the dominant component. (**B**,**C**) The high-risk subgroup exhibited the highest BCR rate and worst prognosis.

Recursive partitioning analysis (RPA) was performed to construct a decision tree to further improve risk stratification for BCR. Based on the pooled cohort, four parameters, namely, GS, pT, SM, and RS, were used as inputs for decision tree construction. Clusters 1–4 (C1–4) with different labels were identified as the outputs of the decision tree. C1 was considered as the low-risk subgroup, C2–3 as intermediate, and C4 as high-risk. The Sankey diagram shows the outcomes of different risk subgroups (Figure 4A). Risk score acted as the dominant factor in the decision tree. Moreover, the low-risk subgroup was labeled with low risk score and negative SM, while the high-risk subgroup was labeled with high risk score and positive SM, further suggesting our signature-derived risk score is the most important factor for risk stratification. Among decision tree-defined subgroups, the high-risk group exhibited the highest BCR rate (Figure 4B) and the worst prognosis (Figure 4C).

### 3.4. Bioinformatic Analyses to Explore Biological Processes Underlying the Gene Signature

**Figure 5:**
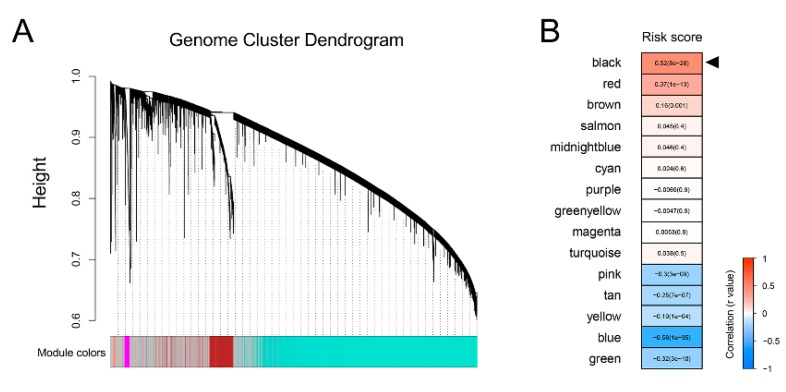

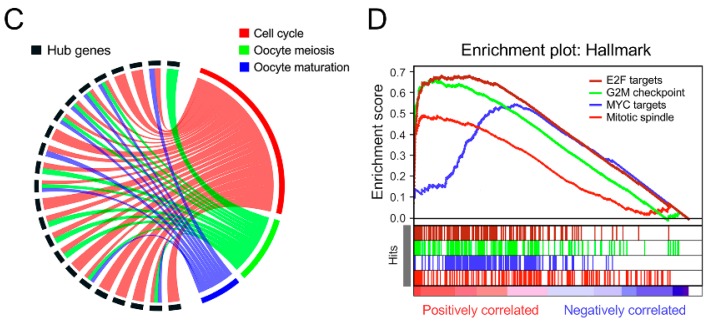
Bioinformatic analyses indicated that the gene signature was correlated with cell cycle-related processes in prostate cancer (PCa). (**A**) WGCNA was performed, and 15 non-grey modules were identified. (**B**) The black module presented the highest correlation with the risk score. (**C**) The Circos diagram showed that hub genes were mainly enriched in cell cycle-related processes. (**D**) GSEA showed that significant predicted signaling pathways were labeled with cell cycle-related hallmarks.

First, sample clustering was performed to exclude outliers (Figure S3). Then, the remaining TCGA samples with RNA-seq data and corresponding risk scores were submitted for WGCNA to construct a scale-free co-expression network. Whole-genome cluster dendrogram trees were generated, and a total of 15 non-grey modules were identified (Figure 5A). A heatmap, as shown in Figure 5B, showed the correlations between the risk score and different modules, and the black module presented the highest correlation with the risk score (r = 0.52, *p* = 8 × 10^−28^). With a threshold of gene significance >0.3, hub genes extracted from the black module were submitted for KEGG enrichment analysis. The Circos diagram showed that hub genes were mainly enriched in terms of “Cell cycle”, “Oocyte meiosis”, and “Oocyte maturation” (Figure 5C). In addition, GSEA was performed to explore potential pathways using low- and high-risk score samples. As shown in Figure 5D, with the “hallmark” gene set, GSEA showed that the significant predicted signaling pathways are: “E2F targets”, “G2M checkpoint”, “MYC targets”, and “Mitotic spindle”.

## 4. Discussion

In recent years, high-throughput transcriptome profiling techniques have been widely applied to identify promising biomarkers for disease diagnosis and prognosis [8,9]. Though some gene signatures have been established to predict the prognosis of PCa patients, few of them have direct relevance to treatment decision-making. In the postoperative setting, the use of adjuvant vs. salvage radiotherapy is until now an unsolved issue permanently under debate. Although three randomized phase III trials demonstrated the benefit of adjuvant radiotherapy in patients with high-risk factors, such as pT3a/b or R1 resection status [4,5,6], about half of these patients will not suffer BCR, for whom adjuvant radiotherapy would be an overtreatment, with an unnecessary risk of radiation-induced side effects. On the other side, salvage radiotherapy is associated with worse prognosis, particularly in patients with high-risk factors, or in patients with high PSA values at initiation of salvage radiotherapy [23,24]. There is a growing body of evidence that the effectivity of salvage radiotherapy is inversely correlated with increases in the salvage treatment PSA [23,25]. Another issue related to the salvage approach is the unclear definition of BCR which should trigger initiation of salvage radiotherapy. Its PSA-value threshold varied from 0.05 to 0.5 in different clinical trials and guidelines [26]. Taken together, optimally, postoperative radiotherapy should be performed in patients who suffer, albeit with possible low PSA values, or in those who are developing BCR with an unmeasurable PSA value at the initiation of radiotherapy. Until now, the prediction of BCR has been based upon clinical parameters, all of them displaying a low predictive accuracy. Thus, any novel biomarkers for a more accurate prediction of BCR would be of high clinical value.

In the present study, we established a nine-gene expression-based signature for BCRFS prediction in PCa patients after prostatectomy and validated it in five other independent datasets, including our own patient cohort. With the transcriptome profiling data and BCRFS status in the training cohort, WGCNA was performed to identify gene modules mostly correlated with BCR, and subsequently, univariate Cox analysis and a LASSO algorithm were applied to overcome overfitting and thus to screen for the most robust biomarkers. Then, the risk score of each patient was calculated with individual normalized expression level and LASSO coefficient according to the established formula. Overall, Kaplan–Meier analysis indicated that patients with higher risk scores exhibited worse BCRFS in each cohort. Moreover, the risk score always serves as an independent risk factor for BCRFS among all the clinicopathological variables in the multivariate Cox regression model. In addition, time-dependent ROC was performed to evaluate the predictive power at different time nodes during follow-up. We observed that the risk score was the most powerful predictor in the GSE70769, GSE70768, and GSE54460 datasets, and was an important predictor beside two clinicopathological features (Gleason score and pT stage) in TCGA, MSKCC, and our cohort. Notably, the risk score was the only significant predictor in all six cohorts, with an extremely stable AUC(t) value of at least 0.75 for each cohort. Furthermore, the prognostic value of the risk score was also validated in the pooled cohort with Z-score normalization.

A decision tree was generated to further optimize risk stratification by combining the risk score with traditional clinicopathological factors. In the pooled cohort-derived decision tree, the risk score functioned as the dominant factor for risk stratification. When stratified by the decision tree, BCRFS varied dramatically in different risk subgroups.

Some biomarkers involved in our gene signature have been investigated in cancer, even in prostate cancer. For instance, *ASNS*, one risk biomarker in our study, was reported to function as a therapeutic target in castration-resistant prostate cancer [27]. *SSTR1* was widely related to the progression of various cancers [28,29,30], and also functions as a prognostic marker in prostate cancer [31,32]. *TRIM14* has been reported to promote invasion in glioblastoma [33] and colorectal cancer [34]. *SRD5A2*, one protective biomarker in our study, inhibits the invasion of prostate cancer cells via regulating the ERK/MAPK pathway [35], and polymorphism in *SRD5A2* contributes to resistance to androgen-deprivation therapy [36]. Regarding *ALDH1A2*, another protective biomarker in our study, it has been reported that the promoter region was significantly hypermethylated in prostate cancer, and overexpression of *ALDH1A2* resulted in decreased colony growth, suggesting that *ALDH1A2* serves as a tumor suppressor in prostate cancer [37]. In addition, *VPS4A* repressed growth and invasion in hepatocellular carcinoma, acting as a tumor suppressor [38]. In a word, the biological roles and clinical significance of the nine genes still need further investigation in PCa.

As our gene signature showed considerable power in risk stratification, the potential biological process and signaling pathways need to be investigated. Using WGCNA co-expression network construction and KEGG enrichment analysis, we observed that the gene signature-related hub genes were mainly enriched in terms of cell cycle. In addition, GSEA indicated that the predicted results that correlated with high risk score were shown as “E2F targets”, “G2M checkpoint”, “MYC targets”, and “Mitotic spindle”, which are also mainly involved in cell cycle-related processes. We suppose that the gene signature-derived cell cycle alteration might contribute to cancer progression and poor prognosis in PCa patients.

Some limitations of our study should be acknowledged. First, this is a retrospective study, so the robustness of the predictive value of the gene signature should be further validated in large prospective clinical trials. Second, experimental studies are required to further elucidate the biological functions underlying the gene signature in PCa.

## 5. Conclusions

In conclusion, we established a gene-expression signature to predict BCRFS in PCa after radical prostatectomy. Integrated with clinicopathological features, a decision tree was generated to further improve the risk stratification for BCR after radical prostatectomy. Our model could be a useful tool for personalized management of PCa patients.

## Supplementary Materials

The following are available online at https://www.mdpi.com/2072-6694/12/1/1/s1, Figure S1: Sample clustering showed no outlier was detected. Figure S2: Expression profiles of the gene signature in primary tumor tissues and adjacent normal tissues from TCGA. Figure S3: Sample clustering was performed to exclude outliers.
