# Supplementary material for: A Novel Gene Signature-Based Model Predicts Biochemical Recurrence-Free Survival in Prostate Cancer Patients after Radical Prostatectomy
Source: Cancers (Basel). 2019 Dec 18;12(1):1. doi: 10.3390/cancers12010001 (PMC7017310; doi:10.3390/cancers12010001)
Supplement: Supplementary file 1 [file cancers-12-00001-s001.pdf]

cancers

Supplementary Materials

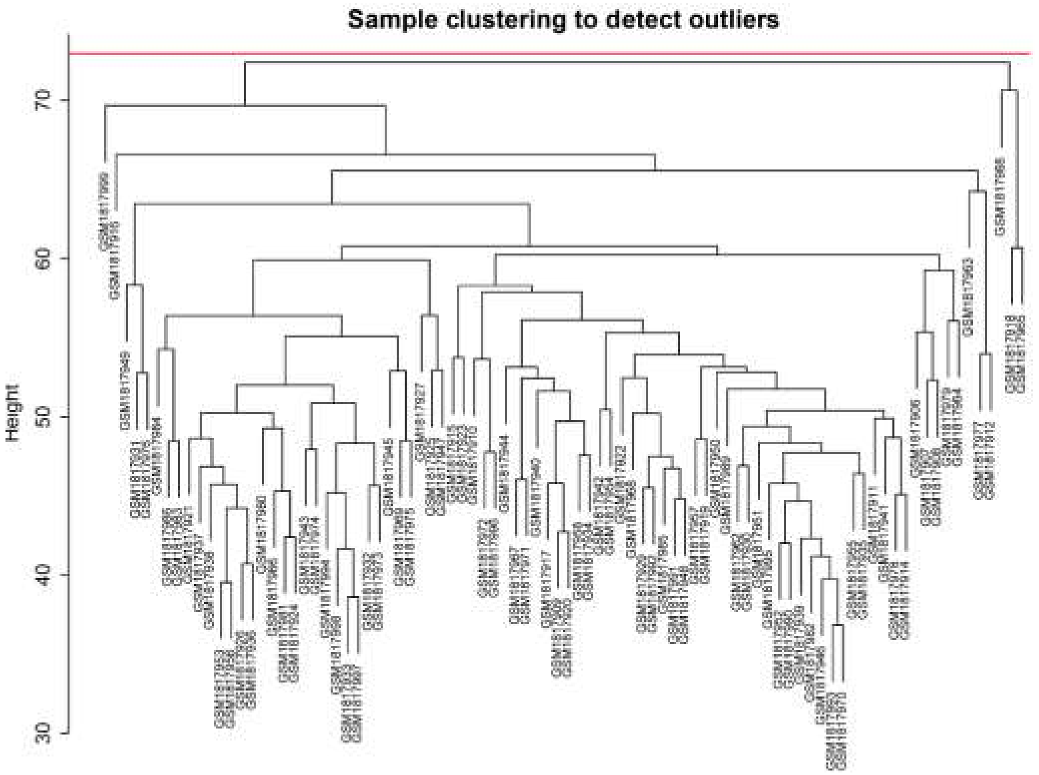

Figure S1. Sample clustering showed no outlier was detected.

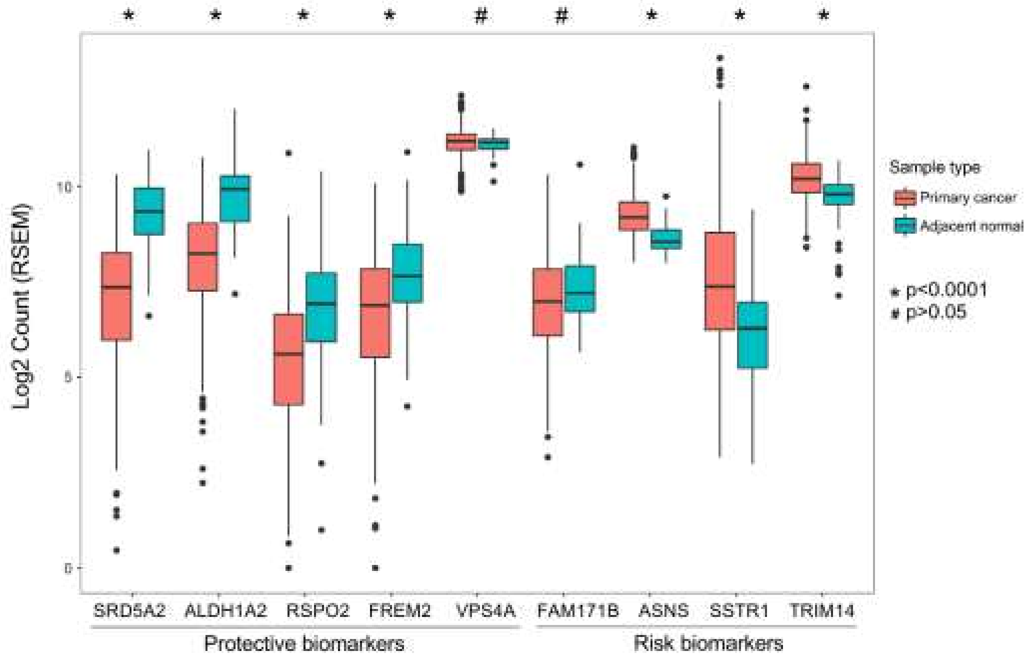

Figure S2. Expression profiles of the gene signature in primary tumor tissues and adjacent normal tissues from TCGA.

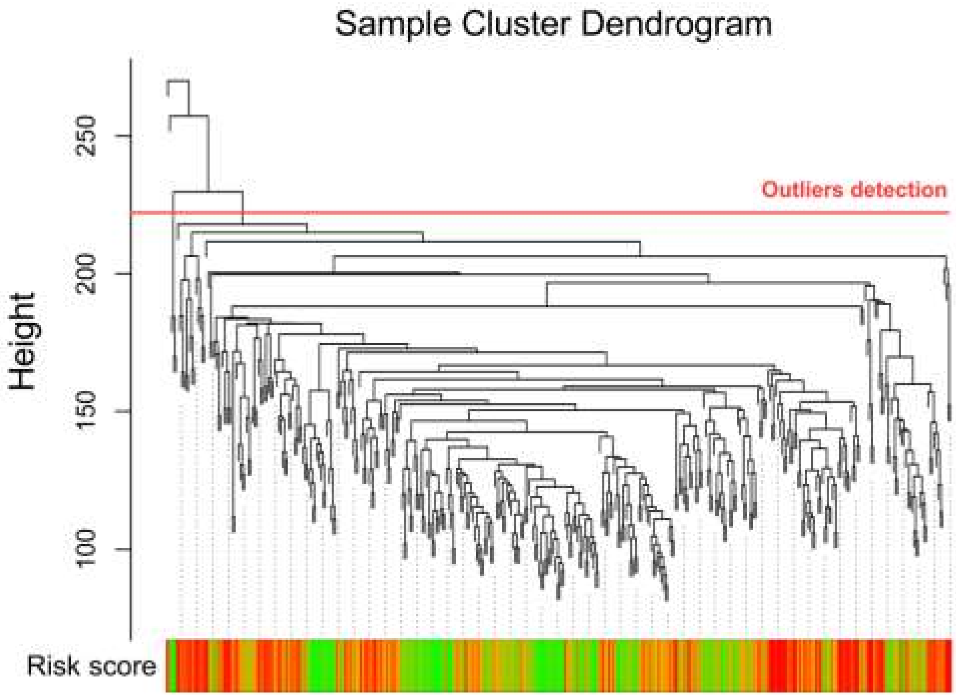

Figure S3. Sample clustering was performed to exclude outliers.
